# Chaldaic Records at Uxmal

**Published:** 1881-11

**Authors:** 


### CHALDAIC RECORDS AT UXMAL.

Dr. de Plongeon, a Mexican antiquarian, announces some interesting discoveries among the ruins of Uxmal, in Yucatan, and he believes that Chaldaic words form an inscription on a stone which, he thinks, forms part of a Masonic lodge. Of the supposed Masonic remains it is not safe to speak until further details are published, but it would not be surprising if, through stone records at the Isthmus, the early civilization of America, now long extinct, were traced to the far East. At present all theories about the source of this civilization must be based on mere conjecture, but as the Mediterranean was full of ships and sailors in the days when the Chaldean language was in daily use in Egypt and Asia Minor, and as the legend of Atlantis, the island in the Western ocean, seems to have existed even at that time, it is not at all improbable that other navigators may have been as thoughtful and venturesome as Columbus. Vessels in the Mediterranean twenty centuries ago are believed to have been quite small, but so were two of the little fleet of Columbus. In short, there would have been nothing more wonderful about an Atlantic voyage in the days of Cæsar, when Carthaginians seem really to have sailed from the Mediterranean to England, than a similar trip in the time of Columbus, whereas, there were good reasons for the lucky discoverers not returning to their native land. Elbow room and the right to do as one chose, has not been easy to find on the Mediterranean within two thousand years. Fac-similes of the Uxmal records will be awaited with great interest.—*N. Y. Herald.*

